# Outcomes of sequential microvascular tissue transfer for radiation induced sequelae post mandibular reconstruction

**DOI:** 10.3389/fonc.2026.1897431

**Published:** 2026-10-23

**Authors:** Karnav Panchal, Sagnika Ukil, Neha Kalwadia, Saugata Samadder, Kapila Manikantan, Pattatheyil Arun, Vikram D. Kekatpure, Prateek V. Jain, Guru Charan Sahu

**Affiliations:** Department of Plastic, Reconstructive and Microsurgery, TATA Medical Center, Kolkata, India

**Keywords:** plate exposure, radiation induced sequelae, SCIP flap, sequential microvascular tissue transfer, superficial temporal vessels

## Abstract

**Background:**

Radiotherapy-related complications following mandibular reconstruction remain a major challenge in head and neck oncologic reconstruction. Although free fibula flap reconstruction has become the gold standard for segmental mandibular defects, delayed radiation-induced sequelae such as hardware exposure, contour deformity, soft tissue atrophy, fistula formation continue to affect long-term outcomes. Literature regarding the role of sequential microvascular tissue transfer in managing these complications remains limited.

**Objectives:**

This study aims to evaluate the indications, surgical planning, recipient vessel selection, and outcomes of sequential microvascular free tissue transfer performed for radiation-induced sequelae after mandibular reconstruction.

**Materials and methods:**

A retrospective observational study was conducted between June 2024 and December 2025 at a tertiary referral center. Patients who had previously undergone primary mandibular reconstruction using a fibula free flap followed by adjuvant radiotherapy and later developed radiation-related complications requiring secondary microvascular reconstruction were included. Demographic data, primary reconstructive details, radiation parameters, duration between radiation and complication, indications for sequential reconstruction, flap characteristics, recipient vessel selection, postoperative complications, and healing outcomes were analyzed.

**Results:**

Ten patients were included in the study. All patients had undergone primary fibula free flap reconstruction following segmental mandibulectomy followed by sequential microvascular soft tissue reconstruction for radiation induced complications. Ipsilateral superficial temporal vessels were used as recipient vessels in all cases without using vein grafts. All flaps survived completely. Satisfactory wound healing and contour restoration were achieved in all patients.

**Conclusion:**

Sequential microvascular reconstruction represents a reliable and effective strategy for managing radiation-induced complications following mandibular reconstruction. Volume loss can be addressed during plate removal surgery which not only achieves a stable soft tissue cover but also prevents further progression to osteoradionecrosis.

## Introduction

Segmental mandibular defects resulting from ablative surgery for advanced head and neck malignancies significantly impair mastication, speech, deglutition, airway protection, and facial aesthetics. Reconstruction of these composite defects has evolved considerably over the past three decades, with vascularized osseous free flaps becoming the standard of reconstruction. Among these, the fibula osteocutaneous free flap remains the workhorse flap ever since its inception due to its long pedicle, reliable anatomy, bi-cortical bone stock, potential for dental rehabilitation, and ability to reconstruct extensive mandibular defects (1).

Radiation therapy plays a critical role in locoregional control of advanced oral cavity malignancies in adjuvant settings; however, it also induces progressive tissue fibrosis, endothelial damage, impaired neovascularization, chronic inflammation, and tissue hypoxia, thereby causing several negative effects, including decreased local tissue vascularity and alteration of the bone-to-metal interface, which increases the risk of plate exposure (2).

The present study evaluates our institutional experience with sequential microvascular soft tissue reconstruction for radiation-induced sequelae following mandibular reconstruction which is not well described in the literature. All the included patients in the study frame had radiation induced sequelae which were evaluated with a CT Imaging to rule out recurrence or osteoradionecrosis. Any suspicious lesion underwent a biopsy to confirm recurrence. All the remaining patient underwent an elective surgery for radiation induced sequelae. We specifically analyzed the interval between the completion of radiotherapy and the secondary surgery, indications for surgery, flap selection, recipient vessel choice, perioperative outcomes, and reconstructive success in this complex subset of patients. The primary objective of the study is to analyze the outcomes of Sequential microvascular tissue transfer for radiation induced sequelae post mandibular reconstruction.

## Materials and methods

This is a retrospective hospital based observational study from June 2024 to December 2025. The inclusion criteria were all follow up patients who had primary mandibular reconstruction by a free fibula flap and have received adjuvant radiotherapy. All these patients had a combination of plate exposure along with associated soft tissue volume loss. The patients who had plate exposure with no or minimal soft tissue volume loss or those patients who were not willing for surgical correction for volume loss were excluded from this study. Patients with established osteoradionecrosis (ORN) were excluded in this study.

All the patient underwent a CT Scan of Head and neck area 3 months post completion of the adjuvant therapy. This was to rule out any recurrence and osteoradionecrosis. Any suspicious lesion underwent a biopsy. They also underwent orthopantomogram (OPG) prior to initiation of adjuvant treatment, 3 months post radiotherapy and prior to planning of the surgery to assess the bony union of the mandibular reconstruct. Data was collected of the initial excision defect, type of primary reconstruction and the pathological staging of the malignancy. The modality, dosage of adjuvant therapy and the interval from the completion of adjuvant therapy to the sequential microvascular tissue transfer was calculated. Data was collected on the indications, type, dimension of flap, selection of recipient vessels, vascular complications and healing related issues.

## Results

Ten patients were included in the study design. Eight were male and two were female. The mean age was 51 years (40–73 years) The pathology was a primary squamous cell carcinoma (SCC) in 9 patients and one patient had a recurrent SCC. Defect was a compound segmental mandibulectomy which were mandibulectomy with buccal mucosa excision in six patients and full thickness segmental mandibulectomy in four patients which were defined as a segmental mandibulectomy along with mucosa and overlying cheek skin defect. Bilateral selective neck dissection was done in seven patients and unilateral in three patients. All the patients underwent a fibula flap reconstruction primarily. Three patients underwent bipaddle fibula and one patient underwent double free flaps which included fibula and superficial circumflex illac artery perforator (SCIP) flap. One patient underwent primary dental implants over the fibula flap. Low profile long plates were used in all the patients for fixation of the fibula reconstruct. The pathology was T4 in all the patients. All of the patients received adjuvant radiotherapy. One patient also received concurrent chemotherapy along with radiation therapy (**Table 1**).

**Table 1:** Demographic data and details of primary surgery with staging.

Sr No	Age/gender	Pathology	Defect of the first surgery	Neck dissection	Primary flap	Plates used	TNM stage
1	49/M	SCC Mandible	Segmental mandibulectomy+ Mucosa	B/L SND	Fibula	Mini plate	T4N0
2	46/M	SCC Mandible	Full thickness segmental mandible	B/L SND	Bipaddle Fibula	Long plate	T4aN0
3	40/F	SCC Mandible	Segmental mandibulectomy+ Mucosa	Left SND	Fibula	Long plate	T4N0
4	62/M	Recurrent SCC Mandible	Full thickness segmental mandible	Right SND	Bipaddle Fibula	Long plate	rT4N0
5	40/M	SCC Mandible	Full thickness segmental mandible	B/L SND	Fibula + SCIP	Long plate	T4N2b
6	56/M	SCC Mandible	Full thickness segmental mandible	B/L SND	Bipaddle Fibula	Long plate	T4aN3b
7	56/M	SCC Mandible	Segmental mandibulectomy+ Mucosa	B/L SND	Fibula	Long plate	T4aN0
8	73/M	SCC Mandible	Segmental mandibulectomy+ Mucosa	Left SND	Fibula	Long plate	T4aN0
9	48/M	SCC Mandible	Segmental mandibulectomy+ Mucosa	B/L SND	Fibula	Long plate	T4aN2b
10	46/F	SCC Mandible	Segmental mandibulectomy+ Mucosa	B/L SND	Fibula	Long plate	T4aN0

None of the patients had trismus prior to the initial surgery. Maximum interincisional opening (MIO) was done for all the patients prior to the initial surgery, post-surgery and after completion of adjuvant radiotherapy. However two patients had developed trismus post radiotherapy(20%) which was defined as MIO less then 25 mm. All the patients had class 1 occlusion and were on solid diet post-surgery. The duration between the completion of adjuvant therapy and secondary surgery ranged from 7 to 60 months, with a mean duration of 21 months. Nine patients underwent sequential reconstruction for plate removal and correction of the volume deficit, while one patient presented with an orocutaneous fistula that required corrective reconstruction. During surgery, the plates were completely removed while strictly avoiding periosteal stripping of both the fibula flap and the native mandible.

All sequential reconstructions utilized soft-tissue free flaps: four anterolateral thigh (ALT) flaps, three radial forearm free flaps (RFFF), and three superficial circumflex iliac artery perforator (SCIP) flaps. Flap selection was guided by patient preference regarding donor-site morbidity and skin-tone matching with the cheek. Because most of these patients had undergone prior bilateral neck dissection, the ipsilateral superficial temporal vessels were used as recipient vessels in all cases. No attempts were made to explore the previously operated and irradiated neck for recipient vessels. All flaps survived without the need for vein grafts or re-explorations. The mean hospital stay was five days, and all patients achieved satisfactory healing without wound complications or volume deficits (**Tables 2**, **3**). For the two patients who underwent secondary surgery at seven months, they also had a uneventful recovery and did not develop any further sequalae. Post the sequential reconstruction all the patients underwent an Orthopantomogram at three months and a CT Scan of the head and neck region at six months duration. Radiological and clinical evaluation were done to rule out any Osteoradionecrosis, recurrence and evaluate the fibula bone.

**Table 2:** Details of sequential surgery with flap and recipient vessels details.

Sr No	Age/gender	Interval between RT completion and sequential reconstruction	Sequential surgery	Flap for sequential reconstruction	Recipient vessels
1	49/M	60 months	Plate removal + volume correction	RAFF	Right Superficial temporal
2	46/M	36 months	Plate removal + volume correction	SCIP	Right Superficial temporal
3	40/F	33 months	Plate removal + volume correction	ALT	Left Superficial temporal
4	62/M	12 months	Plate removal + volume correction	ALT	Left Superficial temporal
5	40/M	17 months	Plate removal + volume correction	ALT	Right superficial temporal
6	56/M	14 months	Plate removal + volume correction	SCIP	Left Superficial temporal
7	56/M	12 months	Plate removal + volume correction	SCIP	Left Superficial temporal
8	73/M	12 months	Plate removal + volume correction	ALT	Left Superficial temporal
9	48/M	7 months	Plate removal + volume correction	RAFF	Right superficial temporal
10	46/F	7 months	Plate removal + OC Fistula correction	RAFF	Right superficial temporal

RAFF, Radial forearm flap; ALT, Anterolateral thigh flap; SCIP, Superficial circumflex iliac artery perforator flap.

**Table 3:** Patient details including pathology, primary defect, sequential flap details.

Sr No	Details	Data
1	Demographic data	Age : 51 years (40 – 73 years Gender : male: 8 female: 2
2	Pathology	Primary Squamous cell carcinoma : 9 Recurrent SCC : 1
3	Defect	Segmental mandibulectomy + Mucosa : 6 Segmental mandibulectomy + Mucosa + Skin: 4
4	Interval (between the completion of adjuvant therapy and secondary surgery)	7 – 60 months
5	Flap for sequential reconstruction	ALT: 4 SCIP: 3 RAFF: 3
6	Recipient vessels	Superficial temporal vessels : 10

Discussion: Late sequelae of radiation therapy are defined as complications appearing more than 90 days after treatment ends (3). One of such late effects is radiation induced fibrosis (RIF), which is the result of a misguided wound healing response. Radiation injury triggers inflammation and stimulates differentiation of fibroblasts into myofibroblasts. These myofibroblasts produce excess collagen and other extracellular matrix (ECM) components, which leads to fibrosis. It manifests as skin induration and thickening, muscle shortening and atrophy and is a major factor in functional and aesthetic impairment in head and neck cancer patients significantly affecting their quality of life. The inflammatory process ultimately evolves into a fibrotic one characterized by increased collagen deposition, poor vascularity, and scarring.

Hardware-related complications remain among the most frequent delayed complications following mandibular reconstruction. Previous studies have shown that titanium can cause a backscatter effect, which may increase the risk of local overdoses around the plate and contribute to screw loosening, osteoradionecrosis, and wound breakdown (4).

Reconstruction plates may be exposed secondary to radiation-induced thinning of the overlying soft tissue envelope, chronic inflammation, infection, or progressive fibrosis (5). Plate exposure is often associated with contour deformity, chronic discharge, pain, and significant psychosocial distress. Furthermore, exposed hardware may predispose patients to secondary osteoradionecrosis involving either the native mandible or the vascularized bone flap. (5This is compounded by volume loss both in the native skin and subcutaneous tissue as well as the flap. Meta-analysis by P Pfister et al. shows flap volume loss 6 months after reconstructive surgery were 24% in irradiated flaps and 18% in nonirradiated flaps. After 1 year, the average total flap volume loss was 38% for the irradiated flaps and 24% for the nonirradiated flaps (6).

In our patient cohort, plate exposure was consistently associated with significant fibrosis of the overlying soft tissue, involving the native skin in six patients and the flap skin island in the four patients who had previously undergone full-thickness reconstruction. All patients experienced concomitant volume loss, which was a major subjective concern. A computed tomography (CT) scan was performed for all patients to rule out tumor recurrence and exclude osteoradionecrosis (ORN). Bone union at the osteotomy and fixation sites were assessed by orthopantomogram Although initial management of plate exposure was conservative and incorporated antibiotic therapy, patients were subsequently offered definitive surgical intervention. This comprised complete plate removal and sequential free tissue transfer to address the volume deficit and replace the radiation-induced, scarred, and non-pliable soft tissue.

However, patients with significant soft tissue loss, chronic plate exposure, fistula formation, or progressive tissue fibrosis frequently require surgical intervention. Local and regional flaps may be inadequate in previously irradiated and vessel-depleted necks due to compromised vascularity and limited tissue availability. Although, microvascular free flap reconstruction has become the reconstruction of choice for advanced ORN, there remains limited data on the management of exposed hardware (7).

Sequential microvascular reconstruction refers to a second free flap procedure performed after an initial successful free tissue transfer in the same anatomical region (8). These procedures are technically demanding due to scarred tissue planes, prior vessel utilization, radiation-induced fibrosis, and limited recipient vessel options. Nevertheless, advances in microsurgical planning and alternative recipient vessel strategies have enabled successful reconstruction even in hostile irradiated fields.

The literature on sequential free tissue transfer for post-radiotherapy complications following mandibular reconstruction remains sparse. Most available studies focus primarily on salvage surgery for recurrent malignancy (9) or osteoradionecrosis (10) rather than contour correction and hardware-related complications after successful primary reconstruction. This article highlights the indications, outcomes and the feasibility of sequential reconstruction in this specific subset of patients. Since the patient are undergoing a surgery for plate removal, correction of soft tissue atrophy post radiotherapy can be corrected in the same sitting with a sequential reconstruction. It also provides additional vascularized tissue to promote healing and prevent further infections.

Use of the superficial temporal artery and vein for scalp and face reconstruction is reliable and safe (11). However, they are still underutilized for lower face defects. With previous neck dissections and previous radiotherapy, the superficial temporal vessels as recipient vessels for sequential reconstruction remains an important alternative. The location being superficial, the dissection is straightforward and predictable and the final scar is well hidden. In our cohort, the superficial temporal vessels were successfully utilized in all patients. No vein grafts were required, and no vascular complications were encountered. Since we routinely use superficial temporal vessels for sequential reconstruction, we avoid using External jugular vein as a recipient vein during the primary surgery.

By providing additional vascularized tissue in a previously operated and irradiated place, it improves the soft tissue equilibrium, achieves primary healing in a hostile environment. Fenske et al. had analyzed prior plate exposure as a key predictor for further flap osteoradionecrosis (12).) If plates and bone gets exposed to there is higher chance of infection and tissue damage. With adjuvant radiotherapy, the healing capacity gets reduced and the patients are more prone for Osteoradionecrosis (12). In our study the patients were followed up for 6 months with CT Scan imaging and OPG. None of our patients developed subsequent ORN. However a longer follow up period is required to come to a conclusion that it would prevent further progression of Osteoradionecrosis.

## Conclusion

Sequential microvascular tissue transfer for radiation induced sequelae promotes healing by bringing vascularized tissue in plate related complications following mandibular reconstruction. This approach not only achieves primary healing but also effectively corrects the associated volume deficit and possibly prevent further progression of the reconstructed bone to ORN. Utilization of alternative recipient vessels such as superficial temporal vessels can simplify sequential microvascular reconstruction in such subsets of patients.

## Data Availability

The original contributions presented in the study are included in the article/supplementary material. Further inquiries can be directed to the corresponding author.
